# Anosognosia for memory deficits in mild cognitive impairment: Insight into the neural mechanism using functional and molecular imaging

**DOI:** 10.1016/j.nicl.2017.05.020

**Published:** 2017-05-25

**Authors:** Patrizia Vannini, Bernard Hanseeuw, Catherine E. Munro, Rebecca E. Amariglio, Gad A. Marshall, Dorene M. Rentz, Alvaro Pascual-Leone, Keith A. Johnson, Reisa A. Sperling

**Affiliations:** a Department of Neurology, Massachusetts General Hospital, Harvard Medical School, Boston, MA 02115, USA; b Athinoula A. Martinos Center for Biomedical Imaging and the Department of Psychiatry, Massachusetts General Hospital, Harvard Medical School, Charlestown, MA 02114, USA; c Center for Alzheimer Research and Treatment, Department of Neurology, Brigham and Women's Hospital, Harvard Medical School, Boston, MA 02115, USA; d Department of Radiology, Division of Molecular Imaging and Nuclear Medicine, Massachusetts General Hospital, Harvard Medical School, Charlestown, MA 02114, USA; e Berenson-Allen Center for Noninvasive Brain Stimulation, and Division for Cognitive Neurology, Beth Israel Deaconess Medical Center, Harvard Medical School, Boston, MA 02115, USA; f Department of Neurology, Saint-Luc University Hospital, Institute of Neuroscience, Université Catholique de Louvain, 1200 Brussels, Belgium

**Keywords:** Alzheimer's disease, Anosognosia, Awareness, Mild cognitive impairment, Magnetic resonance imaging, Positron emission tomography

## Abstract

Anosognosia, or loss of insight of memory deficits, is a common and striking symptom in Alzheimer's disease (AD). Previous findings in AD dementia patients suggest that anosognosia is due to both functional metabolic changes within cortical midline structures involved in self-referential processes, as well as functional disconnection between these regions. The present study aims to extend these findings by investigating the neural correlates of anosognosia in the prodromal stage of AD. Here, we used regional brain metabolism (resting state 18-F fluorodeoxyglucose positron emission tomography (FDG-PET)) to unravel the metabolic correlates of anosognosia in subjects with amnestic mild cognitive impairment (aMCI) and subsequently resting state functional magnetic resonance imaging (rs-fMRI) to investigate the intrinsic connectivity disruption between brain regions. Thirty-one subjects (mean age: 74.1; Clinical Dementia Rating (CDR) global score: 0.5) with aMCI, and 251 cognitively normal (CN) older adults (mean age: 73.3; CDR: 0) were included as a reference group for behavioral and FDG data. An anosognosia index was obtained by calculating a discrepancy score between subjective and objective memory scores. All subjects underwent FDG-PET for glucose metabolism measurement, and aMCI subjects underwent additional rs-fMRI for intrinsic connectivity measurement. Voxel-wise correlations between anosognosia and neuroimaging data were conducted in the aMCI subjects. Subjects with aMCI had significantly decreased memory awareness as compared to the CN older adults. Greater anosognosia in aMCI subjects was associated with reduced glucose metabolism in the posterior cingulate (PCC) cortices and hippocampus. Intrinsic connectivity analyses revealed a significant association between anosognosia and attenuated functional connectivity between the PCC seed region and orbitofrontal cortex (OFC) as well as bilateral inferior parietal lobes (IPL). These findings provide further evidence that implicates cortical midline structures and hippocampus in the awareness of memory deficits. Investigating neuroimaging changes that co-vary with memory awareness may improve our ability to identify the cause of anosognosia and ultimately increase our chances for its treatment.

## Highlights

• A multimodal approach to study the mechanism underlying anosognosia is proposed.
• Mild Cognitive Impairment subjects underestimate their memory deficits.
• Anosognosia is related to reduced posterior cingulate and hippocampal metabolism.
• Anosognosia is related to disconnection between regions subserving self-reflection.

## 1 Introduction

Impaired awareness of memory deficits, a.k.a. anosognosia, is a challenging manifestation of Alzheimer's disease (AD) that adversely affects the patient's safety and decision making and often imposes a negative impact on caregiver quality of life. Anosognosia is a common feature at the AD dementia stage (Agnew and Morris, 1998), but its prevalence in mild cognitive impairment (MCI), considered to be the prodromal stage of dementia, remains uncertain (Roberts et al., 2009). Some studies report MCI patients have intact insight into their cognitive difficulties (Kalbe et al., 2005), while other studies have reported a severe lack of insight (Galeone et al., 2011). Understanding the pathophysiological mechanism underlying impaired awareness of memory deficits in the prodromal stage could help discriminate individuals who are likely to progress to AD dementia from those individuals whose altered awareness of memory is not associated with an underlying pathophysiology.

Although there has been extensive work on the behavioral characterization of anosognosia in AD, (as summarized in a review by Kaszniak and Edmonds (2010)), our knowledge of the neural mechanisms underlying anosognosia remains imprecise and there is no clear-cut consensus on a specific localization of anosognosia. In fact, while anosognosia is a frequent finding in AD dementia, to date relatively few studies have investigated the neuroanatomical correlates of anosognosia in AD dementia (Harwood et al., 2005, Marshall et al., 2004, Ott et al., 1996b, Perrotin et al., 2015, Reed et al., 1993, Ruby et al., 2009, Salmon et al., 2006, Starkstein et al., 1995) and MCI (Ries et al., 2007, Ries et al., 2012, Vogel et al., 2005). The most common and consistent finding across these studies is the association between anosognosia and dysfunction in prefrontal, including dorsolateral and orbitofrontal regions, as well as in the temporoparietal regions, inferior parietal cortex, posterior cingulate cortex and precuneus. The observation that these findings overlap with brain regions which have been implicated in self-referential processing in normal individuals (Johnson and Ries, 2010, Northoff and Bermpohl, 2004, Northoff et al., 2006, Schmitz and Johnson, 2007), has led to the proposal that anosognosia may be caused (in part) by the alteration of internally oriented self-related processes (Ott et al., 1996a).

In support for this hypothesis, Perrotin and colleagues recently demonstrated in a group of 23 patients diagnosed with probable AD that anosognosia was related to both functional metabolic changes within cortical midline structures as well as functional disconnections between these cortical midline regions (Perrotin et al., 2015). Similarly, Ries and colleagues found, in a mixed group of 12 AD and MCI patients, that individuals with anosognosia showed reduced functional connectivity between the medial prefrontal cortex (PFC) and proximal areas within the medial PFC, dorsolateral PFC and left hippocampus (Ries et al., 2012).

The present study aims at extending these findings by further our understanding of the brain mechanism underlying anosognosia in the prodromal stage of AD. Thus, we used a multimodal approach consisting of two steps: First, we used regional brain metabolism (18-F fluorodeoxyglucose positron emission tomography (FDG-PET)) to unravel the metabolic correlates of anosognosia in patients with MCI. Thereafter, we used resting state fMRI (rs-fMRI) to investigate the intrinsic connectivity disruption between brain regions using the hypometabolic FDG brain regions as seed regions in our resting state maps. We expected to find functional metabolic changes in brain regions critical for self-assessment and hypothesized that decreased awareness of memory deficits would be related to disruption between these brain regions.

## 2 Material and methods

### 2.1 Subjects

All subjects were enrolled in an investigator-initiated study at Brigham and Women's hospital (BWH) and Massachusetts General Hospital (MGH) in Boston, Massachusetts. Thirty-one patients with amnestic MCI (single or multiple domain) were included in the present study. They all had a mini-mental state exam (MMSE) (Folstein et al., 1975) score of 24–30 (inclusive), a Clinical Dementia Rating (CDR) (Morris, 1993) global score of 0.5 (with memory box score of 0.5 or 1), essentially preserved instrumental activities of daily living (as determined by a clinician), and no evidence of dementia. Of note, subjects with non-amnestic MCI were not eligible to participate in this study.

Two hundred and fifty-one healthy older adults were included as a reference group. To be included as a cognitively healthy older subject, participants had to have an MMSE score of 27–30 (inclusive with educational adjustment), a global CDR score of 0, and perform within normal range on all neuropsychological tests.

Demographic and clinical features of patients and controls are detailed in Table 1.

**Table 1:** Demographic and clinical variables.

	CN	MCI	Statistical test	*p*-value
N	251	31		
CDR	0	0.5		
Age, y	73.3 (6.2)	74.3 (7.9)	*t* = − 0.81	0.42
Gender, female %	58.9	45.2	χ^2^ = 2.2	0.18
Education, y	15.7 (3.1)	15.7 (3.1)	*t* = 8.11	0.88
AMNART	120.6 (9.4)	119.0 (12.0)	*t* = 0.89	0.37
MMSE	28.9 (1.1)	27.1 (1.9)	*t* = 5.38	< 0.001
Objective memory	13.6 (3.4)	6.2 (5.4)	*t* = 7.5	< 0.001
Subjective memory	5.2 (0.9)	4.4 (0.9)	*t* = 4.8	< 0.001
Memory awareness	0.07 (1.1)	− 0.8 (1.3)	*t* = 3.9	< 0.001

Mean and standard deviation (SD). AMNART = American National Adult Reading Test; CDR = clinical dementia rating; CN = cognitively normal; MCI = Mild cognitive impairment, MMSE = Mini-Mental State Examination; y = years.

#### 2.1.1 Exclusion criteria and consent

Exclusion criteria for all subjects included history of neurologic or major psychiatric disorder, history of head trauma with loss of consciousness, contra-indications for MRI scanning, use of medications that affect cognitive function, severe cardiovascular disease, alcohol or substance abuse, or known cerebrovascular disease (as determined by a Hachinski Ischemia Score (Rosen et al., 1980) higher than 4 and/or presence of cortical infarct, multiple lacunar strokes, or extensive white matter hyperintensities on structural MRI).

This study was approved by and conducted under the auspices of the Partners Human Research Committee at the BWH and MGH (Boston, MA). Informed written consent was obtained from every subject prior to experimental procedures.

### 2.2 Neuropsychological testing

#### 2.2.1 Objective memory

The Logical Memory IIa of the Wechsler Memory Scale-Revised (Wechsler, 1987) is a standardized test that assesses immediate and delayed free recall of a short story, consisting of 25 bits of information. The 20 min delayed free recall score was used as the objective memory score for this study, with lower scores indicating greater memory impairment.

#### 2.2.2 Subjective memory

The “general frequency of forgetting” subscale of the Memory Functioning Questionnaire (Gilewski et al., 1990) was used to assess self-reported memory decline. We used the 18 first questions of this subscale, which contains items regarding everyday situations in which a person would need to use his/her memory. The scale requires participants to rate their memory in terms of the kind of problems they experience on a 7-point scale ranging from 1 (‘major problems’) to 7 (‘no problem’). The scale was transformed to numbers ranging from 0 to 6 and the sum of all 18 questions was used as the subjective memory measure for this study, with lower score indicating greater memory difficulties.

#### 2.2.3 Estimation of awareness of memory deficits

Self-awareness was assessed by calculating the discrepancy scores between the objective and subjective measures of memory function. In short, raw scores from objective and subjective measures were converted to z-scores for each subject, using the mean and SD from our control group as the reference group. A delta score was computed by subtracting the subjective memory score from the objective memory score. By this convention, a greater negative value indicates decreased self-awareness of memory functioning or more severe anosognosia.

### 2.3 Neuroimaging protocols

#### 2.3.1 FDG-PET

FDG was acquired for 45–75 min after a 5 to 10 mCi bolus injection in 6 × 5-min frames. To evaluate the anatomy of PET binding, each individual PET data set was rigidly co-registered to the subject's MRI data using SPM8 (Wellcome Department of Cognitive Neurology, London). Additional preprocessing steps included spatial normalization to the Montreal Neurological Institute (MNI) space (resampled voxel size 2x2x2mm) using the parameters estimated from the corresponding T1-weighted MRI; and quantitative scaling using the cerebellum grey matter as reference to obtain standardized uptake volume ratio images. The resulting images were used in the correlation analyses with the memory awareness score.

#### 2.3.2 Resting state fMRI

All resting-state fMRI data were collected at the Athinoula A. Martinos Center for biomedical imaging in Charlestown, MA on two matched 3 T Trio Tim scanners (Siemens Medical Systems, Erlangen, Germany) using 12-channel phased-array head coils. Scanner noise was attenuated using foam earplugs, and subjects were instructed to lie still, remain awake, and keep eyes open. A functional gradient echo-planar pulse sequence, covering the whole-brain, including the cerebellum, was acquired in the coronal plane using the following parameters: TR = 2500 ms, TE = 30 ms, and FA = 90°, 3.0 mm isotropic voxels. 148 volumes were acquired in each of two 6:10 min runs (including 4 dummy volumes: 10 s). All resting state data were processed using SPM8 (http://www.fil.ion.ucl.ac.uk/spm/). The first four volumes of each run were excluded to allow for T1 equilibration. Each run was slice-time corrected, realigned to the first volume of each run with INRIAlign (http://www-sop.inria.fr/epidaure/software/INRIAlign/; (Freire and Mangin, 2001)), normalized to the MNI 152 EPI template (Montreal Neurological Institute, Montreal, Canada), and smoothed with a 6 mm FWHM Gaussian kernel. Following these standard preprocessing steps, additional processing known to be beneficial for fcMRI analysis was conducted. These included: (1) regression of realignment parameters (plus first derivatives) to reduce movement artifacts on connectivity, (2) temporal band-pass filtering (second order Butterworth filter) to remove frequencies outside of the 0.01–0.08 Hz band, and (3) nuisance regressors (and their first derivatives) for mean signal from the white matter, lateral ventricles, and global brain signals, with motion parameters were applied (Van Dijk et al., 2010). For each subject, we created seed-based correlation maps using a 10-mm-diameter spherical seed, located on the maxima of activation identified by the FDG analysis.

### 2.4 Statistics

Whole brain voxel-wise SPM t correlation maps were used to examine memory awareness (used as a continuous variable), as a predictor of cerebral glucose metabolic rate (CMR_glc_) metabolism in the MCI patients. Maps were thresholded using a cluster-level FDR correction < 0.05, corresponding to a *p* = 0.001, k = 115. This threshold was derived using the method described in Chumbley et al. (2010). Resulting clusters were used as seed regions in the intrinsic connectivity analysis. Thus, for each of the two seeds obtained from the FDG analysis (right hippocampus and precuneus, see blue areas in Figs. 3A and B), we created one seed-based connectivity map per subject. We then computed a one-sample *t*-test map (qFDR < 0.05), investigating which voxels from the whole brain were functionally connected with the seed (Figs. 3A and B: orange areas). Finally, we regressed the memory awareness score in the connectivity maps (*p* < 0.050, Figs. 3A and B: white areas), restricting the results to the voxels significantly connected with the seed, used the resulting map from the previous step, i.e. orange areas, as a mask. Age was entered as a covariate and controlled for in all analyses.

## 3 Results

### 3.1 Participants

No significant differences were found between CN and aMCI subjects on all demographic variable, except for MMSE scores (Table 1). In the aMCI subjects, no significant relationship was found between the memory awareness score and age (*p* = 0.7); gender (*p* = 0.4), or education (*p* = 0.7). A significant positive relationship was found between memory awareness score and MMSE (*r* = 0.5, *p* = 0.009) indicating that decreased awareness (anosognosia) was related to worse global cognition.

Significant group differences were found for objective and subjective memory scores as well as for the memory awareness score. In comparison to the CN, the aMCI patients had lower objective delayed free recall scores, more subjective memory complaints and decreased memory awareness discrepancy scores (i.e. anosognosia or unaware of their memory deficits; Table 1).

### 3.2 Relationship between glucose metabolism and anosognosia in MCI patients

**Fig. 1:**
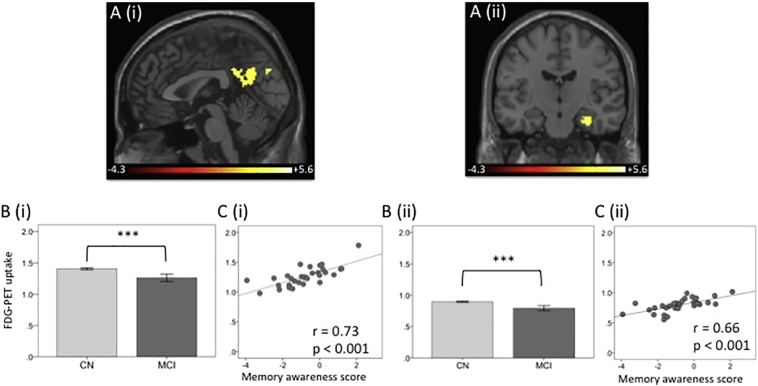
(A) Voxel-wise statistical parametric maps of the significant correlations between FDG-PET uptake and memory awareness discrepancy score in (i) precuneus and (ii) right hippocampus. Maps are controlled at pFDR < 0.05 using age as covariate. Color bar shows the t-statistics. (B) Bar graphs of the healthy cognitively normal (CN) and mild cognitive impairment (MCI) subjects group comparison on the FDG uptake values in (i) precuneus and (ii) hippocampus. *** = *p* < 0.001. (C) Plot of the correlations between FDG uptake in (i) precuneus and (ii) hippocampus and the memory awareness discrepancy score in MCI patients. *P*-values are 2-tailed. Lower score represent less awareness (anosognosia).

The whole brain voxel-wise analysis, entering memory awareness discrepancy score as a continuous variable revealed two significant clusters: bilateral posterior midline regions (cluster size: 742) with a peak in precuneus, BA 7 (MNI coordinates: x = 8, y = − 74, z = 32); and right medial temporal lobe (MTL) including hippocampus (cluster size: 115) with a peak in parahippocampus (MNI coordinates: x = 24, y = − 10, z = − 26) (Fig. 1A).

Post hoc analysis revealed significantly reduced glucose metabolism in the MCI patients as compared to CN individuals (indicating that these areas are hypometabolic in MCI patients) (Fig. 1B). In the MCI patients, a significant positive correlation between memory awareness and glucose metabolism was found, such that decreased awareness (anosognosia) was related to lower glucose metabolism in these regions (Fig. 1C).

### 3.3 Reduced intrinsic connectivity with Anosognosia in MCI patients

**Fig. 2:**
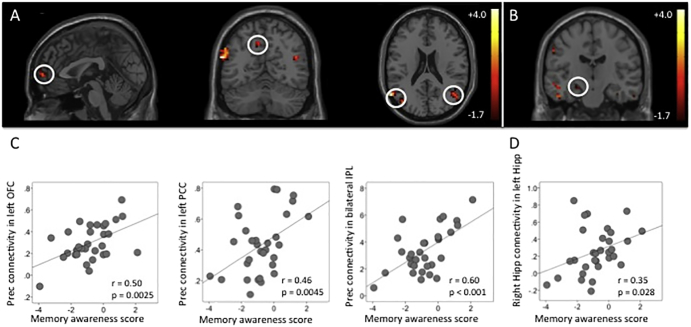
(A) Intrinsic connectivity maps (*p* = 0.05, cluster extent = 5, controlling for age) between the precuneus seed region and the orbitofrontal cortex (OFC), left posterior cingulate cortex (PCC), and bilateral inferior parietal loves (IPL; circled) and between Color bar shows the t-statistics. (B) right hippocampus seed region and the left medial temporal lobe including hippocampus (circled). (C) Plot of the correlation between the intrinsic connectivity using the precuneus as seed region in the significant clusters and the memory awareness score. (D) Plot of the correlation between the intrinsic connectivity using the right hippocampus as seed region in the significant clusters. *P*-values are two-sided. Lower score represent decreased awareness (anosognosia).

**Fig. 3:**
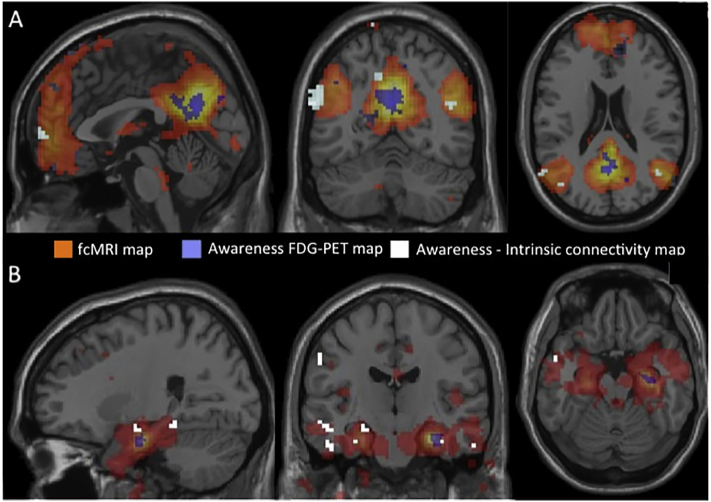
Overlay of the awareness-FDG-PET and awareness-intrinsic connectivity maps (color coded in white and purple) on top of the intrinsic connectivity map for the (A) PCC seed region respectively (B) MTL seed region elicited in the MCI patients (orange). (For interpretation of the references to color in this figure legend, the reader is referred to the web version of this article.)

To investigate whether functional connectivity of a network involving the precuneus and hippocampus was related to anosognosia in our MCI patients, we used the hypometabolic brain regions we had found with FDG-PET as our seed regions and entered memory awareness as a covariate (see Fig. 3A, purple area). Using the FDG-PET precuneus seed in the intrinsic connectivity analysis (*p* < 0.05, cluster extent = 5 voxels, controlling for age), we found significant positive correlations between the bilateral inferior parietal lobes (IPL); left posterior cingulate cortex (PCC); and the orbitofrontal cortex (OFC). In all these regions, decreased memory awareness was significantly related to decreased functional connectivity (Fig. 2A and C). Using the FDG-PET right hippocampus seed in the intrinsic connectivity analysis (*p* < 0.05, cluster extent = 5 voxels, controlling for age; Fig. 3B, purple area), we found significant correlations between several brain regions in the left MTL, as well as in right fusiform gyrus. In all these regions, decreased memory awareness was significantly related to decreased functional connectivity (Fig. 2B and D).

## 4 Discussion

The present study investigated the brain mechanism underlying unawareness of memory deficits by combining measures of regional brain metabolism (as measured with FDG-PET) and intrinsic connectivity (as measured with resting state fMRI). Our results demonstrate that decreased awareness of memory deficits (anosognosia) in aMCI subjects is related to FDG hypometabolism in precuneus and hippocampus. Furthermore, using the hypometabolic regions as seed regions, we found that functional disconnections between cortical midline regions were related to increased anosognosia. These results provide further evidence and support the hypothesis that anosognosia may be the result of both functional metabolic changes and functional disconnection between brain regions supporting self-referential processing as well as memory processing.

Our findings of decreased awareness of memory deficits in aMCI, as compared to CN, provide further evidence supporting the finding that anosognosia is present in the prodromal stages of AD (Roberts et al., 2009). Further, our finding of a positive relationship between memory awareness and MMSE score is in line with previous studies of AD patients which indicate that anosognosia correlates with overall disease severity (Kalbe et al., 2005).

The results of our voxel-wise correlations between the memory awareness discrepancy score and FDG-PET data were consistent with our hypothesis to find functional metabolic changes in brain regions critical for self-assessment. FDG-PET imaging is suggested to directly reflect neural activity at rest and provides an index of functional integrity (Sokoloff et al., 1977). Thus, our findings of decreased glucose metabolism in the precuneus and hippocampus, which was hypometabolic relative to the CN sample, provides further evidence that anosognosia is in part due to compromise to brain regions critical for retrieval and manipulation of autobiographical episodic memories, and more recently self-referential processing. (Andrews-Hanna et al., 2010, Whitfield-Gabrieli et al., 2011). For instance, previous work investigating successful retrieval has shown greater activation in the posteromedial cortices in proportion to the strength or certainty of the memory decision (Svoboda et al., 2006, Wagner et al., 2005). Similarly, previous studies have shown that these regions are involved in subjective judgments about memory retrieval, including “remember vs. know” assessments and metamemory processes, such as postretrieval confidence ratings (Chua et al., 2006, Wheeler and Buckner, 2004).

In a fMRI study, Ries et al. (2007) reported decreased neuronal activation during a self-referencing task in the posterior cingulate and medial prefrontal cortex in MCI subjects who were less aware of their own deficits. Our results are in line with Perrotin et al. (2015), who found that anosognosia in AD dementia patients was associated with hypometabolism (using FDG-PET) in posterior cingulate cortices. These results also converge with our recent work investigating the metabolic correlates of subjective memory complaints in cognitively normal older adults. Using a similar voxel-wise approach we entered subjective memory complaints into whole brain correlation maps and found that increased complaints were related to decreased glucose metabolism in the cortical midline regions (Vannini et al., 2017b). Since these individuals were cognitively normal at the time of testing, their memory complaints would indicate heightened awareness of their memory ability (Vannini et al., 2017a). Indeed, it has recently been proposed by our group (Vannini et al., 2017a), as well as by Dalla Barba et al. (2015), that the level of self-awareness varies in a continuum from normal awareness of cognitive performance to anosognosia through a phase in which the individual experiences awareness of normal performance as impaired. Our findings of reduced hippocampal metabolism in individuals with increased anosognosia are somewhat concurrent with previous studies demonstrating decreased hippocampal metabolism in prodromal AD (Hanseeuw et al., 2016, Mosconi et al., 2008). Interestingly, in our previous study we found that cognitively normal individuals with increased cortical amyloid burden and increased complaints (heightened awareness) had decreased FDG metabolism in the hippocampus, providing further evidence that the functional disturbance of this region may be a specific marker of changes in awareness encompassing both the preclinical and prodromal stage of AD (Vannini et al., 2017b).

Furthermore, and in line with Perrotin et al. (2015), by using resting state fMRI and the PCC as seed regions, we found a significant association between anosognosia and attenuated functional connectivity between the PCC seed region and OFC, as well as bilateral IPL. These results provide further evidence that disconnection between the PCC and OFC may reflect a disruption of the white matter fiber pathways making up the cingulum bundle (Villain et al., 2010). In contrast to Perrotin et al.’s work, we did not find a relationship between our PCC seed region and the medial temporal lobe. This discrepancy might be due to the fact that Perrotin and colleagues investigated individuals with AD dementia who are likely to be further along the pathological trajectory of AD. Future studies looking at the either the baseline difference between MCI and AD dementia patients or at longitudinal changes within subjects on the trajectory of AD may further clarify the pathophysiology underlying anosognosia at different stages of the disease. Nonetheless, the findings of disconnection within the cortical midline regions support the hypothesis that anosognosia is caused by alteration of internally oriented self-related processes (Schneider et al., 2008). Thus, it can be argued that the disconnection with the self-referential regions might impair the metamnemonic monitoring processes necessary to update the person's knowledge about their memory skills.

In contrast to our expectations as well as with previous studies, such as (Perrotin et al., 2015), we did not find a reduced functional connectivity between hippocampus and the OFC or PCC. We did, however, find reduced functional connectivity between the left and right hippocampus (using the FDG-PET right hippocampus as our seed region) in individuals with reduced awareness. Previous studies investigating hippocampal connectivity in AD dementia patients have reported diminished hippocampal connectivity in AD dementia patients when compared to healthy controls who instead present rightward asymmetry between the two hippocampi (Wang et al., 2010). Future studies on the specific relationship between hippocampal connectivity and the relationship with awareness are needed to disentangle these results. Also, inclusion of a sample with AD dementia patients might shed some further light on this.

We acknowledge that there are several limitations to this study. First, there is no consensus on how to optimally assess anosognosia in AD (see Starkstein (2014)). The primary methods being (1) clinical judgment, (2) comparison of study partner to informant reports, and (3) comparison of participants' self-assessment and task performance. Here we used the third approach, which has been suggested to present an advantage in that it is comparing a subjective memory report to an objective gold standard. As this method was used by Perrotin et al. (2015), we also opted for this approach to be able to more easily compare our results to their results. Future studies will utilize other subjective memory questionnaires to investigate potential discrepancies between the subject's self-report and the report of the informant to get further insight into the self-awareness of these memory problems in our participants and how that is related to FDG metabolism and functional connectivity. In addition, we acknowledge that this was a cross-sectional study and that longitudinal studies will be necessary to determine whether our findings may serve as a relevant predictor of AD progression.

## 5 Conclusions

By combining measures of regional brain metabolism (as measured with FDG-PET), and intrinsic connectivity (as measured with resting state fMRI), we found that decreased awareness of memory deficits (anosognosia) in aMCI patients may be the result of both functional metabolic changes, as well as a functional disconnection between brain regions supporting self-referential processing and memory processing. Investigating neuroimaging changes that co-vary with memory awareness may improve our ability to identify and understand the cause of anosognosia and ultimately increase our chances for its treatment.

## Acknowlewdgement

This research was carried out in whole or in part at the Athinoula A. Martinos Center for Biomedical Imaging at the Massachusetts General Hospital, using resources provided by the *Center for Functional Neuroimaging Technologies, P41EB015896*, a P41 Regional Resource supported by the National Institute of Biomedical Imaging and Bioengineering (NIBIB), National Institutes of Health. P. Vannini received funding from NIH-NIA grant K01AG048287. B. Hanseeuw was supported by Belgian-American Education Foundation.

Sperling receives research support from NIH grants R01 AG037497, R01 AG034556, and K24 AG035007. The content is solely the responsibility of the authors and does not necessarily represent the official views of the National Institutes of Health.

## Disclosure

P. Vannini received funding from NIH-NIA grant K01AG048287.

B. Hanseeuw was supported by Belgian-American Education Foundation.

C. Munro reports no disclosures relevant to the manuscript.

R. Amariglio was supported by Alzheimer's Association NIRG-12-243,012 and NIH grant K23AG044431. She is coinvestigator for Eisai, Eli Lilly, and Merck.

G. Marshall receives research support for NIH grant K23 AG033634. G. Marshall has served as paid consultant for Halloran Consulting Group and Grifols. He is a site principal investigator coinvestigator for Janssen Alzheimer Immunotherapy, Wyeth/Pfizer Pharmaceuticals, Eisai Inc., Eli Lilly and Company, Avid Radiopharmaceuticals, Bristol-Myers-Squibb, Merck, and Navidea clinical trials. These relationships are not related to the content in the manuscript.

D. Rentz received research support from the NIH grants P01 AG036694, R01MH090291, U01 AG024904, R01 AG027435, R01 AG037497, and P50 AG005134, a Fidelity Investigator-Initiated grant, and Alzheimer Association grant SGCOG-13-282,201. She is coinvestigator for Eli Lilly.

K. Johnson has served as paid consultant for Bayer, Biogen Idec, Bristol-Myers Squibb, GE Healthcare, Isis Pharmaceuticals Inc., Janssen Alzheimer's Immunotherapy, Piramal, Siemens Medical Solutions, and Genzyme. He is a site principal investigator or coinvestigator for Lilly/Avid, Biogen Idec, Bristol-Myers Squibb, Eisai, Pfizer, Janssen Alzheimer Immunotherapy, Merck, and Navidea clinical trials. He has spoken at symposia sponsored by Janssen Alzheimer's Immunotherapy, GEHC, Lundbeck, and Pfizer. These relationships are not related to the content in the manuscript. K. Johnson receives research support from NIH grants R01EB014894, R21, AG038994, R01 AG026484, R01 AG034556, P50 AG00513421, U19 AG10483, P01 AG036694, R13 AG042201174210, R01, AG027435, and R01 AG037497.

A. Pascual-Leone serves on the scientific advisory boards for Nexstim, Neuronix, Starlab Neuroscience, Neuroelectrics, Axilum Robotics, Magstim Inc., and Neosync; and is listed as an inventor on several issued and pending patents on the real-time integration of transcranial magnetic stimulation with electroencephalography and magnetic resonance imaging. A. Pascual-Leone receives research support from NIH grants R01MH100186, R01HD069776, R01NS073601, R21 NS082870, R21 MH099196, R21 NS085491, R21 HD07616, Harvard Catalyst | The Harvard Clinical and Translational Science Center (NCRR and the NCATS NIH, UL1 RR025758), Sidney Baer Jr. Foundation, Michael J. Fox Foundation, and the Football Players Health Study at Harvard University.

R. Sperling has served as a paid consultant for Bristol-Myers Squibb, Eisai, Janssen Alzheimer Immunotherapy, Pfizer, Merck, and Roche, and as an unpaid consultant to Avid and Eli Lilly. She is a site principal investigator or coinvestigator for Avid, Bristol-Myers Squibb, Pfizer, and Janssen Alzheimer Immunotherapy clinical trials. She has spoken at symposia sponsored by Eli Lilly, Pfizer, and Janssen Alzheimer Immunotherapy. These relationships are not related to the content in the manuscript. R. Sperling receives research support from NIH grants U01 AG032438, U01 AG024904, R01 AG037497, R01 AG034556, K24 AG035007, P50 AG005134, U19 AG010483, R01 AG027435, and P01 AG036694.
